# Supplementary material for: Prevalence, Management, and Associated Factors of Obesity, Hypertension, and Diabetes in Tibetan Population Compared with China Overall
Source: Int J Environ Res Public Health. 2022 Jul 19;19(14):8787. doi: 10.3390/ijerph19148787 (PMC9316255; doi:10.3390/ijerph19148787)
Supplement: Supplementary file 1 [file ijerph-19-08787-s001.zip › ijerph-1761997-supplementary.pdf]

Supplementary Material

1 Supplementary Tables and Figures

1.1 Supplementary Tables

Supplementary Table S1. Characteristics and key findings of the included studies for systematic review (n=39) and meta-analysis (n=37).

References	Country/area	Study setting and survey year	Study subjects	Study outcome assessment	Overweight/obesity (%)	Hypertension (%)	Diabetes (%)	Study quality*
**A Studies reported prevalence of all three outcomes**								
1.Meng QT, 2018	Sichuan	Community- and Buddhist institute - based;<br>2016-2017	n=2182<br>Buddhists n=1384<br>M (664, 48.0), F (720, 52.0)<br>Age: 45.1±15.6<br>Adult residents n=798<br>M (367, 46.0), F (431, 54.0)<br>Age: 45.8±15.4	Ov/ob: Chinese criteria; HTN; DM	Ov/ob:<br>Buddhists (All 51.4, M 52.6, F 50.3);<br>Adult residents (All 49.3, M 53.7, F 45.6)	Buddhists (All 23.3, M 26.2, F 20.6);<br>Adult residents (All 41.0, M 47.1, F 35.7)	Buddhists (All 1.7, M 1.7, F 1.6);<br>Adult residents (All 8.7, M 10.7, F 7.6)	Good
2.Peng Y, 2018	Changdu, TAR;<br>Alt: 3500m	Hospital-based;<br>2016-2017	n=1253<br>Tibetans adults<br>M (597, 47.6), F (656, 52.4)<br>Age: mean 52, range 30-94	Ov/ob: Chinese criteria; HTN; DM	Ov/ob: 53.6	All 49.8, M 51.2, F 48.5;<br><br>By Alt<br>3159-3500m 47.6;<br>3700-4200m 62.7	27.0	Fair
3.Xu SP, 2015	Changdu, TAR;<br>Alt: 3200-4500m	Community- and Buddhist institute-based;<br>2010-2011	n=1659<br>U (395, 23.8), R (936, 56.4), Buddhist institute (328, 19.8)<br>M (822, 49.5), F (837, 50.5)<br>Age ≥ 18	Ov/ob: Chinese criteria; HTN; DM	Ov/ob:<br>All 35.6, M 41.1, F 30.1;<br><br>By Alt<br><3500m 42.6; ≥3500m 27.1;<br>By residence<br>U 50.4; R 25.5; Buddhist institute 46.9	All 64.6, M 64.1, F 65.1;<br><br>By Alt<br><3500m 68.7; ≥3500m 59.7;<br>By residence<br>U 70.1; R 67.2; Buddhist institute 50.6	All 6.8, M 7.7, F 5.9;<br><br>By Alt<br><3500m 8.4; ≥3500m 5.0;<br>By residence<br>U 12.5; R 5.7;<br>Buddhist institute 2.6;	Good
4.Okumiya K, 2015	Qinghai, China and Ladakh, India;<br>Alt: 2900-4800m	Community-based;<br>2008-2011	n=1258<br>U (632, 50.2), R (626, 49.8)<br>M (541, 43.0), F (717, 57.0)	Ov/ob: WHO criteria; HTN; DM	Ov/ob:<br>All 40.1, M 36.6, F 42.8;<br>By residence<br>U 55.6; R 24.6	All 40.4, M 42.7, F 38.7;<br><br>By residence	All 8.9;<br><br>By residence<br>U 9.5; R 8.3	Fair

			Age: 58.0±11.5, 40-87			U 48.8; R 31.9		
5.Sherpa LY, 2013	Lhasa, TAR; Alt: 3700m	Community-based; 2010	n=692<br>R residents (623, 90.0)<br>M (317, 45.8), F (375, 54.2)<br>Age: ≥ 30	Central ob: IDF criteria; HTN; DM	Central ob (All 46.0, M 25.0, F 64.3)	All 32.5, M 32.8, F 32.2	All 3.9, M 2.5, F 5.0	Fair
6.Chen W, 2010	Lhasa and Dangxiong, TAR; Alt: 3658-4200m	Community-based; NR	n=1289<br>Tibetans adults<br>Age: 43.7±14.3	Ov/ob: Chinese criteria; Central ob: IDF criteria; HTN; DM	Ob (All 16.7, M 19.3, F 14.2)<br>Central ob (All 41.2, M 30.6, F 51.7)	All 38.8, M 45.1, F 32.1	All 2.9, M 3.1, F 2.5	Fair
**B Studies reported prevalence of two types of outcomes**								
7.Chen YM, 2020	Gannan, Gansu; Alt: 3000-4900m	Kindergartens-based; 2018-2019	n=2364<br>Preschoolers<br>M (1226, 51.9), F (1138, 48.1)<br>Age: 3.9±1.2	Ov/ob: WHO criteria in 2006; HTN	Ov/ob (All 2.3, M 2.7, F 1.8)	All 4.6, M 6.1, F 2.9	NR	Fair
8.Deng, RD, 2020	TAR	Based on the 2018 Sixth National Health Service Survey of TAR; 2018	n=10493<br>Tibetans adults<br>U (2186, 20.8), R (8307, 79.2)<br>M (4921, 46.9), F (5572, 53.1)<br>Age: 44.1±15.7	HTN; DM	NR	14.4	0.9	Fair
9.Ci RWM, 2020	Lhasa, TAR	Community-based; 2016-2017	n=1798<br>U (1390, 77.3), R (408, 22.7)<br>M (582, 32.4), F (1216, 67.6)<br>Age: ≥18	Ov/ob: Chinese criteria; Central ob: WGOC criteria; HTN	Ov/ob (All 60.9, M 76.2, F 53.6)<br>Central ob (All 41.8, M 51.7, F 37.0)	All 25.7, M 35.1, F 21.2	NR	Fair
10.Zhang H, 2020	Lhasa, TAR	Community-based; 2017	n=1053<br>Tibetans adults<br>M: (390, 37.0), F: (663, 63.0)<br>Age: 47.9±13.5, ≥18	Ob: Chinese criteria; HTN	Ob (All 31.4, M 31.5, F 31.4)	All 30.7, M 37.9, F 26.4	NR	Fair
11.Huang YS, 2019	Diqing, Yunnan	Community-based; 2012-2013	n=1379<br>Tibetans adults	Ov/ob: Chinese criteria; Central ob: WGOC criteria; HTN	Ov/ob 45.6<br>Central ob 39.4	35.0	NR	Poor

12.Sun PF, 2019	Lhasa, TAR	Community-based; NR	n=1486<br>Tibetans adults<br>M (548, 36.9), F (938, 63.1)<br>Age: ≥18	HTN; DM	NR	27.0	3.3	Fair
13.Ye ZZ, 2018	Tianzhu and Gannan, Gansu;<br>Alt: 1100-4900m;<br>Lhasa and Shigatse, TAR;<br>Alt: 3650-3860m	Community-based; 2014-2016	n=916<br>U (322, 35.2), R (594, 64.8)<br>M (469, 51.2), F(447, 48.8)<br>Age: 46.2±12.1, 18-82<br>Alt: ≥3000m (407, 44.4), <3000m (509, 55.6)	Ob: Chinese criteria; HTN	Ob 30.7	All 45.0, M 41.4, F 48.8;<br><br>By Alt<br>≥3000m 41.4; <3000m 39.9;<br>By residence<br>U 75.5; R 28.5	NR	Fair
14.Liu K, 2018	Ganzi, Sichuan	Buddhist institute-based;<br>2013-2014	n=594<br>Buddhists<br>M (310, 52.2), F (284, 47.8)<br>Age: 41.9±15.5, >18	HTN; DM	NR	19.4	2.7	Good
15.Li K, 2017	Lhasa, TAR	Hospital-based; 2009-2011	n=5478<br>Tibetans undergoing check-up<br>M (3296, 60.2), F (2182, 39.8)<br>Age: 41.9±13.9	Ov/ob: CDS Criteria; HTN	Ov/ob (All 40.2, M 45.0, F 33.8)	All 24.4, M 28.0, F 18.9	NR	Poor
16.Huang XB, 2016	Sichuan;<br>Alt: 2500–4000m	Community-based; 2013-2014	n=3230<br>U (2174, 67.3), R (1056, 32.7)<br>M (1503, 46.5), F (1727, 53.5)<br>Age: mean 43.78, range 15-92	Ov/ob: WHO criteria; Central ob: WGOC criteria; HTN	Ov/ob 17.8<br>Central ob 13.9	All 45.7, M 47.4, F 45.7;<br><br>By residence<br>U 48.0; R 41.0	NR	Fair
17.Zhuoma C, 2015	Xiahe, Gansu;<br>Alt: 3700m	Community-based; 2012-2013	n=1069<br>U (293, 27.4), R (776, 72.6)<br>M (407, 38.1), F (662, 61.9)<br>Age: ≥18	Ov/ob: WHO criteria; HTN	Ov/ob 40.0	All 34.1, M 45.5, F 27.0;<br><br>By residence<br>U 26.3; R 37.0	NR	Fair
18.Li XH, 2015	Gannan Tibetan Autonomous Prefecture, Gansu;<br>Alt: 3000m.	Community- and Buddhist institute-based;<br>2014	n=2026<br>Buddhists n=984<br>Age: 41.7±9.2, 18-70<br>Adult residents n=1042<br>Age: 42.0±8.8, 18-70	Ov/ob: Chinese criteria; HTN	Ov/ob:<br>Buddhists 21.5<br>Adult residents 34.8	Buddhists 19.3<br>Adult residents 34.1	NR	Fair

19.Zhu CK, 2014	Yushu and Guoluo, Qinghai	Hospital-based; 2009-2011	n=25491<br>R residents (Pastoralists)<br>M (15230, 59.7), Age: 18-77, 49.4±6.8<br>F (10261, 40.3), Age: 18-81, 54.2±8.7	HTN; DM	NR	All 38.6, M 42.6, F 32.7	22.4	Fair
20.Zheng X, 2013	Lhasa, TAR; Alt: 3650m	Community-based; 2010	n=1370<br>U (875, 63.9), R (495, 36.1)<br>M (545, 39.8), F (825, 60.2)<br>Age: ≥18	Ov/ob: WHO criteria; HTN	Ov/ob 20.1	All 51.2, M 56.0, F 48.0;<br><br>By residence<br>U 55.1; R 44.2	NR	Good
21.Lai SR, 2011	Nyingchi, TAR; Alt: 3000m	School-based; 2010	n=2777<br>Tibetan students<br>M (1302, 46.9), F (1475, 53.1)<br>Age: 7-18	Ov/ob: WGOC criteria in 2005; HTN	Ov/ob:<br>All 13.9, M 7.2, F 19.8	All (n=1712) 2.9,<br>M (n=871) 3.4,<br>F (n=841) 2.3	NR	Poor
**C Studies reported one outcome**								
**(a) Obesity**								
22.Chen XM, 2020	Lhasa, TAR	School-based; 2019	n=1246<br>Middle school students<br>M (642, 51.5), F (604, 48.5)<br>Age: 14.2±1.7	Ov/ob: WGOC criteria in 2004	Ov/ob (All 15.9, M 20.4, F 11.3)	NR	NR	Good
23.Li TX, 2020	Aba, Sichuan	Community-based; 2018-2019	n=2216<br>U (1102, 49.7), R (1114, 50.3)<br>M (1307, 59.0), F (909, 41.0)<br>Age: 45.0±14.0, 18-80	Ov/ob: NR; Central ob: IDF criteria	Ov/ob 37.7<br><br>Central ob:<br>All 46.3, M 59.6, F 27.2;<br>By residence<br>U 44.8; R 49.2	NR	NR	Fair
24.Shi CL, 2020	Aba and Ganzi , Sichuan	School-based; 2018	n=1921<br>Tibetan students<br>Age: 7-18	Ov/ob: WGOC criteria in 2004	Ov/ob (All 16.6, M 15.4, F 17.8)	NR	NR	Poor
25.Peng W, 2020	Golmud, Qinghai	Urbanized settled Tibetan community- and school-based; 2018	n=1431<br>Adults n=927<br>M (422, 45.5), F (505, 54.5)<br>Age: 43.3±13.9, 18-90<br><br>Children n=504<br>M (244, 48.4), F (260, 51.6)	Ov/ob:<br>For child: WHO criteria in 2007<br>For Adult: Chinese criteria<br><br>Central ob for Adult: WGOC criteria	Ov/ob:<br>Children (All 24.0, M 23.4, F 24.6);<br>Adults (All 61.4, M 61.6, F 61.2)<br><br>Central ob:<br>Adults (All 62.0, M 59.2, F 64.4)	NR	NR	Fair

			Age: 9.4±2.2, 5.8-15.9					
26.Zhang ML, 2019	Changdu, TAR; Alt: 3000-4000m	School-based; 2015	n=979<br>Tibetan students<br>M (530, 54.1), F (449, 45.9)<br>Age: 6-14	Ov/ob: WHO criteria in 2007	Ov/ob (All 21.8, M 21.9, F 21.7)	NR	NR	Fair
27.Xu RB, 2017	Lhasa, TAR	School-based: data from six waves of CNSSCH; Latest 2014	n=2418<br>Tibetan Students<br>M (1214, 50.2), F (1204, 49.8)<br>Age: 7-18	Ov/ob: Chinese criteria for child in 1985	Ov/ob (All 16.4, M 18.5, F 14.2)	NR	NR	Poor
28.Yao YL, 2010	Yushu, Qinghai	Hospital-based; NR	n=690<br>Tibetan civil servants<br>M (458, 66.4), F (232, 33.6)<br>Age: 38.0±14.0, 17-75	Central ob: IDF criteria	Central ob (All 40.6, M 32.9, F 55.6)	NR	NR	Fair
**(b) Hypertension**								
29.Lu CL, 2020	Nagqu, TAR; Alt: 4700-5100m	Community-based; 2020	n=729<br>R residents (Pastoralists)<br>M (290, 39.8), F (439, 60.2)<br>Age: 18-87	HTN	NR	All 13.6, M 18.7, F 10.2	NR	Poor
30.Song C, 2020	TAR:<br>Bomi, Nyingchi; Alt: 2700-3035m;<br>Dagze, Lhasa; Alt: 3719-3970m;<br>Nagarze, Lhokha; Alt: 4462-4505m	Community-based; 2017	n=1631<br>R residents<br>M (614, 37.6), F (1017, 62.4)<br>Age: 20-80<br>Alt 2700-3035m (542, 33.2), 3719-3970m (543, 33.3), 4462-4505m (546, 33.5)	HTN	NR	All 40.3;<br><br>By Alt<br>2700-3035m 53.0;<br>3719-3970m 42.0;<br>4462-4505m 26.2	NR	Good
31.Xu T, 2016	Sichuan and Yunnan	Community-based; 2007-2011	n=885<br>Tibetans adults<br>Age: 18-79	HTN	NR	15.6	NR	Fair

32.Liao AX, 2015	Qiongjie, TAR; Alt: 3700m	Community-based; 2014	n=15243<br>R residents (Farmers)<br>M (7432, 48.8), F (7811, 51.2)<br>Age: ≥18	HTN	NR	All 9.7, M 13.0, F 6.6	NR	Fair
33.Xu T, 2015	Sichuan and Yunnan	Community-based; 2007-2011	n=867<br>Tibetans children and adolescents<br>Age: 8-18	HTN	NR	1.5	NR	Fair
34.Pan MQ, 2014	Liangshan, Sichuan;<br>Low Alt: 1500-2500m, High Alt: 3000-4000m	Community-based; NR	n=2590<br>Low Alt (1261, 48.7), high Alt (1329, 51.3)<br>Age: 30-70	HTN	NR	All 8.4;<br><br>By Alt<br>Low Alt 14.3; High Alt 2.2	NR	Fair
35.Li XH, 2012	Gannan, Gansu; Altitude: 3000 m	Community-based; 2010	n=6948<br>U (3340, 48.1), R (3608, 51.9)<br>M (4246, 61.1), F (2702, 38.9)<br>Age: ≥18	HTN	NR	All 24.6, M 25.0, F 23.9;<br><br>By residence<br>U 25.3; R 23.8	NR	Good
**(c) Diabetes**								
36.Li YZ, 2020	China	Community-based, national representative survey (TIDE study);<br>2015-2017	n=2034<br>Tibetans adults<br>Age: ≥18	DM and Pre-DM	NR	NR	DM 6.5<br>Pre-DM 34.4	Fair
37.Zhang ZX, 2019	Lhasa, TAR;<br>Altitude: 3680m	Community-based; 2017-2018	n=2603<br>U (1816, 69.8), R (787, 30.2)<br>M (951, 36.5), F (1652, 63.5)<br>Age: 42.9±15.8	DM and Pre-DM	NR	NR	DM 4.4<br>By residence<br>U 4.2; R 4.8<br><br>Pre-DM 7.0<br>By residence<br>U 5.6; R 10.2	Fair
38.Chen N, 2019	Lhasa, TAR	Community-based; NR	n=1379<br>Tibetans adults<br>M (899, 65.2), F (480, 34.8)<br>Age: 43.9± 15.0, 18-75	Pre-DM	NR	NR	Pre-DM (All 8.1, M 5.1, F 13.7)	Fair

39.Wang LM, 2017	China	Community-based, national representative survey (CCDRFS study); 2013	n=3103 Tibetans adults M (1328, 42.8), F (1775, 57.2) Age: ≥18	DM and Pre-DM	NR	NR	DM (All 4.3, M 4.7, F 3.9)<br><br>Pre-DM (All 31.3, M 31.8, F 30.9)	Fair

Abbreviations: ADA: American Diabetes Association; Alt: Altitude; BAZ: z-scores of body mass index-for-age; BMI: Body mass index; CCDRFS: China Chronic Disease and Risk Factors Surveillance; CDS: Chinese Diabetes Society; CNSSCH: Chinese National Survey on Students Constitution and Health; DBP: Diastolic blood pressure; ESC: European Society of Cardiology; ESH: European Society of Hypertension; F: Female; FPG: Fasting plasma glucose; IDF: International Diabetes Federation; JNC: Joint National Committee; M: Male; NR: Not report; Ob: Obesity; OGTT: Oral Glucose Tolerance Test; Ov: Overweight; R: Rural; SD: Standard deviation; SBP: Systolic blood pressure; TAR: Tibet Autonomous Region; TIDE: Thyroid disorders, Iodine status and Diabetes Epidemiological; U: Urban; WC: Waist circumference; WGOC: Working Group on Obesity in China; WHO: World Health Organization.
Classification of overweight and obesity: Chinese criteria: overweight (24.0 ≤ BMI < 28.0 kg/m²), obesity (BMI ≥ 28.0 kg/m²); WHO criteria: overweight (25.0 ≤ BMI < 30.0 kg/m²), obesity (BMI ≥ 30.0 kg/m²); CDS Criteria: overweight and/or obesity (BMI ≥ 25.0 kg/m²); WGOC criteria in 2004/2005 for child: Chinese sex-age-specific BMI cutoff points for individuals aged 7–17 years; WHO criteria in 2006 for child: overweight (85th percentile of sex-age-specific BMI ≤ BMI＜95th percentile of sex-age-specific BMI, Obesity (≥ 95th percentile of sex-age-specific BMI); WHO criteria in 2007 for child: overweight (1 < BAZ ≤ 2), obesity (BAZ > 2); Chinese criteria for child in 1985: Take the 80th percentile weight of the same height group as the standard weight, overweight (10% ≤ the standard weight≤ 20%), obesity (≥ 20% of the standard weight).
Classification of central obesity: IDF criteria: men (WC ≥ 90 cm), women (WC ≥ 80 cm); WGOC criteria: men (WC ≥ 85 cm), women (WC ≥ 80 cm).
Classification of hypertension: Chinese Guidelines for the Management of Hypertension (2009 Basic Edition/ 2010/2018 revised edition) ;The JNC 7 criteria/The JNC 8 criteria; ESH/ESC guidelines; CDS Criteria; 2007 European Society of Cardiology guidelines for the management of arterial hypertension: SBP ≥ 140 mmHg and/or DBP ≥ 90 mmHg, or use of antihypertensive medication or self-reported diagnosis of high blood pressure and use of antihypertensive medication; Chinese criteria for Adolescents: average SBP and/or DBP levels ≥ 95% of the same sex, age and height.
Classification of diabetes and pre-diabetes: Chinese Guidelines for the Management of Type 2 Diabetes; ADA criteria; WHO criteria: diabetes (self-reported diabetes and/or FPG ≥ 7.0 mmol/L and/or two-hour plasma glucose ≥ 11.1 mmol/L), pre-diabetes (among participants without diabetes: FPG 5.6 mmol/L to 6.9 mmol/L and/or OGTT: two-hour plasma glucose 7.8 mmol/L to 11.0 mmol/L.
*: Quality assessment for studies is shown in Supplementary Table 2.

Supplementary Table S2. Study quality assessment for the included 39 studies.

Studies	1	2	3	4	5	6	7	8	9	10	11	12	13	14	Total	Quality rating
1. Meng QT; 2018	Yes	Yes	Yes	Yes	No	No	No	NA	Yes	No	Yes	NA	NA	Yes	7	Good
2. Peng Y; 2018	Yes	Yes	NR	Yes	No	No	No	NA	Yes	No	Yes	NA	NA	No	5	Fair
3. Xu, SP; 2015	Yes	Yes	Yes	Yes	No	No	No	Yes	Yes	No	Yes	NA	NA	Yes	8	Good
4. Okumiya K; 2015	Yes	Yes	NR	No	No	No	No	Yes	Yes	No	Yes	NA	NA	Yes	6	Fair
5. Sherpa LY; 2013	Yes	Yes	Yes	Yes	No	No	No	NA	NA	No	Yes	NA	NA	No	5	Fair
6. Chen W; 2010	Yes	Yes	Yes	Yes	No	No	No	NA	NA	No	Yes	NA	NA	No	5	Fair
7. Chen YM; 2020	Yes	Yes	NR	Yes	No	No	No	NA	NA	No	Yes	NA	NA	No	4	Fair
8. Deng, RD; 2020	Yes	Yes	Yes	Yes	No	No	No	NA	NA	No	No	NA	NA	No	4	Fair
9. Ci RWM; 2020	Yes	Yes	NR	Yes	No	No	No	NA	NA	No	Yes	NA	NA	No	4	Fair
10. Zhang H; 2020	Yes	Yes	Yes	Yes	No	No	No	NA	NA	No	Yes	NA	NA	No	5	Fair
11. Huang YS; 2019	Yes	Yes	NR	No	No	No	No	NA	NA	No	Yes	NA	NA	No	3	Poor
12. Sun PF; 2019	Yes	Yes	Yes	Yes	No	No	No	NA	NA	No	Yes	NA	NA	No	5	Fair
13. Ye ZZ; 2018	Yes	Yes	NR	No	No	No	No	NA	Yes	No	Yes	NA	NA	No	4	Fair
14. Liu, K; 2018	Yes	Yes	Yes	Yes	No	No	No	Yes	Yes	No	Yes	NA	NA	Yes	8	Good
15. Li K; 2017	Yes	Yes	NR	No	No	No	No	NA	NA	No	Yes	NA	NA	No	3	Poor
16. Huang XB; 2016	Yes	Yes	Yes	Yes	No	No	No	Yes	No	No	Yes	NA	NA	No	6	Fair
17. Zhuoma C; 2015	Yes	Yes	NR	No	No	No	No	Yes	Yes	No	Yes	NA	NA	No	5	Fair
18. Li, XH; 2015	Yes	Yes	Yes	No	No	No	No	Yes	Yes	No	Yes	NA	NA	No	6	Fair
19. Zhu CK; 2014	Yes	Yes	NR	No	No	No	No	NA	Yes	No	Yes	NA	NA	No	4	Fair
20. Zheng X; 2013	Yes	Yes	Yes	Yes	No	No	No	Yes	Yes	No	Yes	NA	NA	No	7	Good
21. Lai SR; 2011	Yes	Yes	NR	No	No	No	No	NA	NA	No	Yes	NA	NA	No	3	Poor
22. Chen XM; 2020	Yes	Yes	Yes	Yes	No	No	No	Yes	Yes	No	Yes	NA	NA	No	7	Good
23. Li TX; 2020	Yes	Yes	NR	Yes	No	No	No	Yes	Yes	No	Yes	NA	NA	No	6	Fair
24. Shi CL; 2020	Yes	Yes	NR	No	No	No	No	NA	NA	No	Yes	NA	NA	No	3	Poor
25. Peng W; 2020	Yes	Yes	Yes	Yes	No	No	No	NA	NA	No	Yes	NA	NA	No	5	Fair
26. Zhang ML; 2019	Yes	Yes	NR	Yes	No	No	No	NA	NA	No	Yes	NA	NA	No	4	Fair
27. Xu RB; 2017	Yes	Yes	NR	No	No	No	No	NA	NA	No	Yes	NA	NA	No	3	Poor
28. Yao YL; 2010	Yes	Yes	Yes	Yes	No	No	No	NA	NA	No	Yes	NA	NA	No	5	Fair
29. Lu CL; 2020	Yes	Yes	Yes	No	No	No	No	NA	NA	No	No	NA	NA	No	3	Poor
30. Song C; 2020	Yes	Yes	Yes	Yes	Yes	No	No	Yes	Yes	No	Yes	NA	NA	Yes	9	Good
31. Xu, T; 2016	Yes	Yes	Yes	Yes	No	No	No	NA	NA	No	Yes	NA	NA	No	5	Fair
32. Liao AX; 2015	Yes	Yes	Yes	No	No	No	No	NA	NA	No	Yes	NA	NA	No	4	Fair
33. Xu, T; 2015	Yes	Yes	NR	Yes	No	No	No	NA	NA	No	Yes	NA	NA	No	4	Fair
34. Pan MQ; 2014	Yes	Yes	NR	No	No	No	No	NA	Yes	No	Yes	NA	NA	No	4	Fair
35. Li, XH; 2012	Yes	Yes	Yes	No	No	No	No	Yes	Yes	No	Yes	NA	NA	Yes	7	Good
36. Li YZ; 2020	Yes	Yes	Yes	Yes	No	No	No	NA	NA	No	Yes	NA	NA	Yes	6	Fair
37. Zhang ZX; 2019	Yes	Yes	NR	Yes	No	No	No	NA	NA	No	Yes	NA	NA	Yes	5	Fair
38. Chen N; 2019	Yes	Yes	Yes	Yes	No	No	No	NA	NA	No	Yes	NA	NA	No	5	Fair
39. Wang LM; 2017	Yes	Yes	Yes	Yes	No	No	No	NA	NA	No	Yes	NA	NA	No	5	Fair

Abbreviations: NR: Not report; NA: Not applicable.
Based on the US National Institute of Health (NIH) recommendation, the quality of each study was assessed according to the following 14 questions. The full score was 14. Total score ≥7 was rated good; 4-7, fair; and <4, poor.

1. Was the research question or objective in this paper clearly stated?
2. Was the study population clearly specified and defined?
3. Was the participation rate of eligible persons at least 50%?
4. Were all the subjects selected or recruited from the same or similar populations (including the same time period)? Were inclusion and exclusion criteria for being in the study prespecified and applied uniformly to all participants?
5. Was a sample size justification, power description, or variance and effect estimates provided?
6. For the analyses in this paper, were the exposure(s) of interest measured prior to the outcome(s) being measured?
7. Was the timeframe sufficient so that one could reasonably expect to see an association between exposure and outcome if it existed?
8. For exposures that can vary in amount or level, did the study examine different levels of the exposure as related to the outcome (e.g., categories of exposure, or exposure measured as continuous variable)?
9. Were the exposure measures (independent variables) clearly defined, valid, reliable, and implemented consistently across all study participants?
10. Was the exposure(s) assessed more than once over time?
11. Were the outcome measures (dependent variables) clearly defined, valid, reliable, and implemented consistently across all study participants?
12. Were the outcome assessors blinded to the exposure status of participants?
13. Was loss to follow-up after baseline 20% or less?
14. Were key potential confounding variables measured and adjusted statistically for their impact on the relationship between exposure(s) and outcome(s)?

Supplementary Table S3. The Egger's test for publication bias of the included studies for overweight/obesity, diabetes and hypertension.

	**Std. eff.**	**Coef.**	**Std. err.**	***t***	***P*>|t|**	**95% CI**
Overweight and obesity (Chinese criteria)	Slope	0.003	0.303	0.02	0.993	(-0.776, 0.781)
	Bias	38.226	25.084	1.52	**0.188**	(-26.255, 102.708)
Overweight and obesity (WHO criteria)	Slope	0.232	0.188	1.23	0.306	(-0.367, 0.831)
	Bias	7.648	21.118	0.36	**0.741**	(-59.559, 74.854)
Diabetes	Slope	0.010	0.025	0.38	0.709	(-0.045, 0.065)
	Bias	13.315	9.106	1.46	**0.172**	(-6.726, 33.356)
Hypertension	Slope	0.139	0.042	3.32	0.003	(0.052, 0.226)
	Bias	16.514	6.466	2.55	0.018	(3.137, 29.891)

Supplementary Table S4. Subgroup meta-analysis of pooled prevalence (%, 95%CI) estimates of overweight/obesity, hypertension and diabetes among Tibetan population based on the included studies.

	**Overweight and obesity**			**Central obesity**			**Hypertension**			**Diabetes**		
Groups	No. of studies	Prevalence (%, 95% CI)	Heterogeneity P value	No. of studies	Prevalence (%, 95% CI)	Heterogeneity P value	No. of studies	Prevalence (%, 95% CI)	Heterogeneity P value	No. of studies	Prevalence (%, 95% CI)	Heterogeneity P value
Adults (≥ 18 years)												
Men	4	57.7 (44.5, 71.0)	**<0.001**	3	39.2 (19.2, 59.1)	**<0.001**	16	38.4 (30.7, 46.0)	**<0.001**	4	4.9 (3.3, 6.6)	**<0.001**
Women	4	48.2 (34.5, 62.0)	**<0.001**	3	48.9 (22.5, 75.3)	**<0.001**	16	32.4 (25.2, 39.6)	**<0.001**	4	4.6 (3.8, 5.4)	**<0.001**
Urban areas	*	*	*	*	*	*	7	49.5 (37.1, 62.0)	**<0.001**	3	8.7 (3.8, 13.7)	**<0.001**
Rural areas	*	*	*	*	*	*	10	33.5 (22.9, 44.1)	**<0.001**	3	6.3 (4.6, 7.9)	0.034
Buddhist institute	*	*	*	*	*	*	4	27.0 (21.5, 32.4)	**<0.001**	3	2.3 (1.6, 3.1)	0.314
Children (<18 years)												
Boys	3	14.3 (6.5, 22.1)	**<0.001**	*	*	*	2	*	*	0	*	*
Girls	3	16.3 (11.0, 21.7)	**<0.001**	*	*	*	2	*	*	0	*	*

The definition of overweight and obesity used Chinese criteria: BMI ≥ 24 kg/m². The definition of central obesity used International Diabetes Federation (IDF) criteria: male waist circumference (WC) ≥90cm, female waist circumference (WC) ≥ 80cm.
*: Total and/or subgroup meta-analysis was not performed because of small number of studies.

2.1 Supplementary Figures

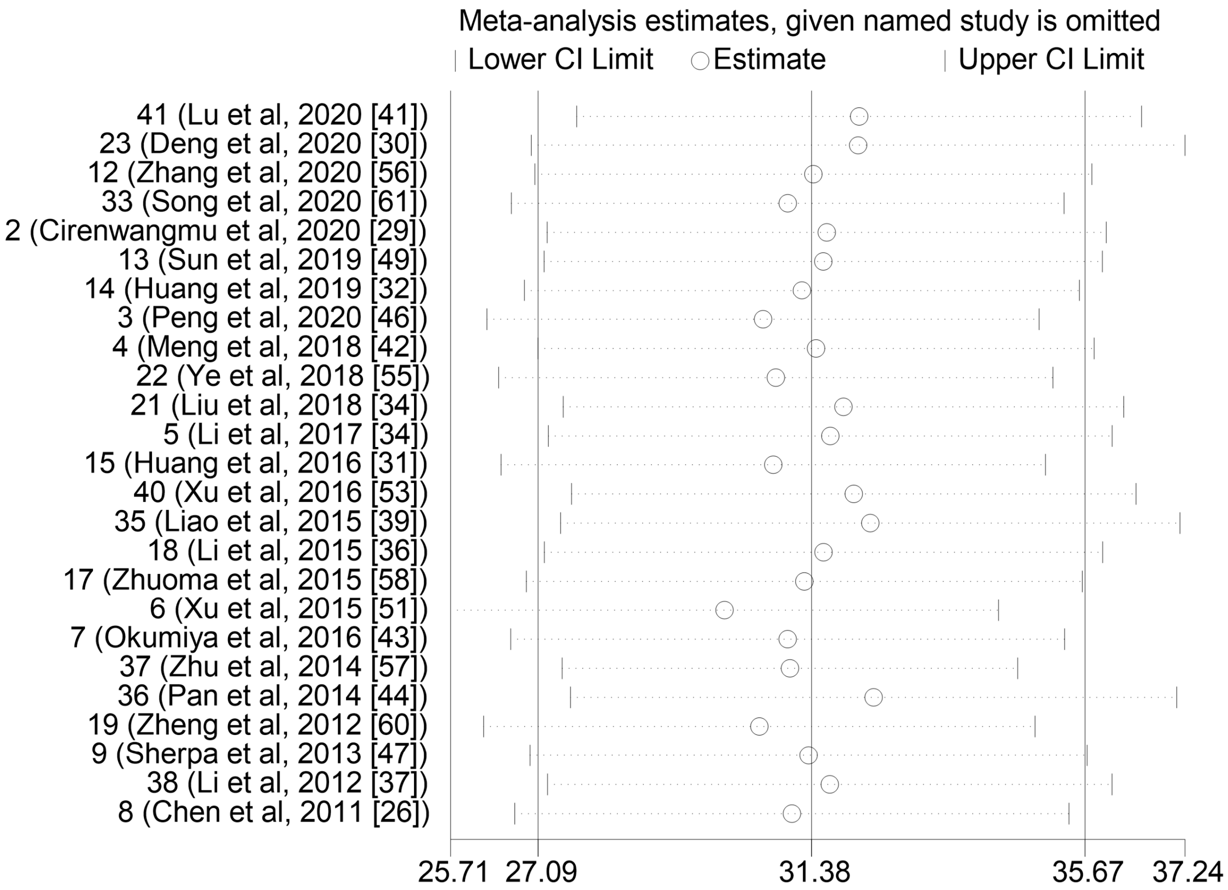

Supplementary Figure S1. Sensitivity analysis of the included studies for hypertension.
